# Kyste hydatique sous cutané isolé: à propos d’un cas et revue de la literature

**DOI:** 10.11604/pamj.2016.24.212.9744

**Published:** 2016-07-12

**Authors:** Bahija Lemrhari, Hasna Baha, Soufia Azzouzi, Soumyia Chiheb

**Affiliations:** 1 Service de Dermatologie, CHU Ibn Rochd, Casablanca, Maroc; 2 Anatomopathologiste du Secteur Privé, Casablanca, Maroc

**Keywords:** Hydatid cyst, solitary, subcutaneous

## Abstract

La localisation sous cutanée, primitive et isolée, du kyste hydatique est exceptionnelle, même dans les pays où la maladie hydatique est endémique. Nous rapportons le cas d’une jeune patiente présentant un kyste hydatique sous cutané sans atteintes viscérales associées.

## Introduction

La maladie hydatique est une infection parasitaire causée par Echinococcus granulosus. Le cycle de vie de ce parasite est bien connu. Le foie est l’organe le plus fréquemment atteint (75%), suivi par le poumon (15%). La localisation primaire dans le tissu sous cutané est extrêmement rare, et son incidence est inconnue. Nous présentons un cas exceptionnel de kyste hydatique sous cutané isolé sans atteintes viscérales associées chez une jeune patiente de 17 ans.

## Patient et observation

**Figure 1:**
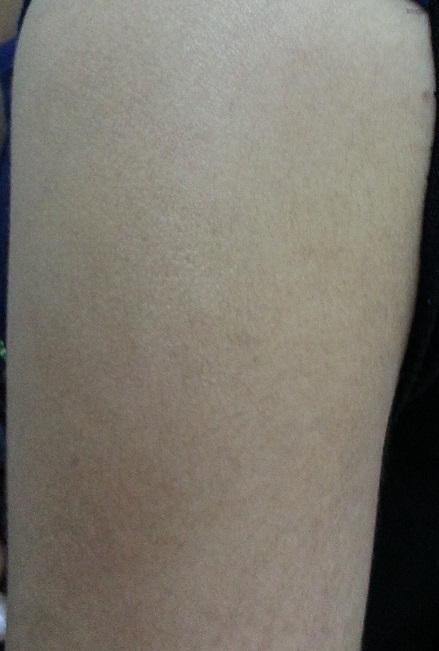
Lésion nodulaire sous-cutanée du bras droit

**Figure 2:**
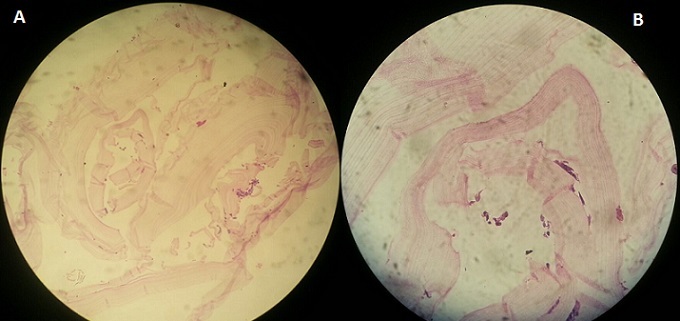
(A) étude histologique montre membrane anhiste feuilletée faite de couches concentriques faiblement éosinophile; (B) une membrane proligère sans anomalie du tissu graisseux

T.K est une patiente âgée de 17 ans sans antécédents pathologiques particuliers qui a présenté depuis deux mois une lésion nodulaire de 1,5 cm de diamètre, mobile au plan profond, douloureuse à la palpation avec une peau normale en regard associée à des céphalées (Figure 1). Le bilan biologique n’avait pas révélé d’hyper éosinophilie. La vitesse de sédimentation était à 18 mm à la première heure. L’exploration chirurgicale avait objectivé une membrane kystique translucide à paroi fine de 1,5cm liée à un tissu graisseux. L’examen histologique avait montré une membrane anhiste feuilletée faite de couches concentriques faiblement éosinophile avec une membrane proligère sans anomalie du tissu graisseux en faveur d’un kyste hydatique sous cutané (Figure 2 A,B). La sérologie hydatique était négative à deux reprises (15 jours d’intervalle). La radiographie du thorax, l’échographie abdominale et la TDM cérébrale étaient normaux. La patiente était mis sous un traitement antiparasitaire albendazole 400mg six jours sur sept avec une surveillance biologique hépatique rigoureuse pendant 06 mois sans récidive ni autres localisations viscérales.

## Discussion

Le mécanisme de la localisation sous cutanée n’est pas clair [1, 2]. Les œufs du parasite ingéré pénètrent la paroi intestinale, rejoignent le système de port et atteignent le foie où la plupart d´entre eux sont pris en charge dans les sinus hépatiques [1, 3]. Quelques œufs peuvent passer à travers le foie (premier filtre) et atteindre le poumon (second filtre) et la circulation systémique provoquant la maladie hydatique dans d´autres organes [4]. Une diffusion possible à travers les canaux lymphatiques a également été rapportée. Ce mécanisme explique les localisations exceptionnelles comme notre cas [2, 3]. La propagation à partir d’un foyer de voisinage constitue un autre mécanisme de dissémination de la maladie hydatique [5]. Dans une grande série grecque de patients, la fréquence des hydatidoses extra-hépatique et extra-pulmonaire était de 9% [2]. Cependant, dans d’autres séries, la fréquence de la localisation sous-cutanée est généralement associée à une atteinte d´autres organes solides et a été estimé à environ 2% [6]. La localisation sous cutanée, primitive et isolée, du kyste hydatique est extrêmement rare, même dans les zones géographiques où la maladie hydatique est fréquente [2, 7]. Habituellement, il se présente comme une masse non-inflammatoire, indolore, sans aucune détérioration de l’état général du patient. Toutefois, si le kyste est infecté ou fissuré, le tableau clinique peut simuler un abcès ou conduire à un choc anaphylactique [6]. L´échographie, la tomodensitométrie et l’IRM sont utiles pour le diagnostic et la recherche d’autres localisations [8]. La sérologie est utile pour confirmer le diagnostic. Elle est rarement positive pour les kystes extra-hépatiques et extra-pulmonaires (25%) [3, 6]. La meilleure option thérapeutique est l’exérèse chirurgicale du kyste intact pour éviter la récidive locale et le risque de choc anaphylactique [6]. Si l’exérèse est impossible, le contenu du kyste peut être aspiré en per opératoire ou sous guidage échographique puis irrigué avec une solution scolicidale: solution saline concentrée à 20%, peroxyde d´hydrogène à 3%, cétrimide à 1,5% associé à la chlorhexidine à 0,15% (10% Savlon^®^), l´alcool éthylique à 95%, la polyvinylpyrrolidone iodée à 10% (Bétadine^®^) [9, 10]. Le traitement médical repose sur l´utilisation de l´albendazole [1, 3].

## Conclusion

Le Kyste hydatique fait partie des diagnostics à évoquer devant toute lésion kystique sous-cutanée, en particulier dans les régions endémiques. La recherche d’autres localisations viscérales est indispensable. Le meilleur traitement est l'excision totale du kyste avec une paroi intacte. Le traitement antiparasitaire avec l’albendazole doit être initié en cas de kyste rompu ou de chirurgie impossible.
